# Online prediction for respiratory movement compensation: a patient-specific gating control for MRI-guided radiotherapy

**DOI:** 10.1186/s13014-023-02341-1

**Published:** 2023-09-11

**Authors:** Yang Li, Zhenjiang Li, Jian Zhu, Baosheng Li, Huazhong Shu, Di Ge

**Affiliations:** 1 Laboratory of Image Science and Technology, School of Computer Science and Engineering, Southeast University, Nanjing, 210096 Jiangsu People’s Republic of China; 2 Department of Radiation Oncology, Shandong Cancer Hospital and Institute, Shandong First Medical University and Shandong Academy of Medical Sciences, Jinan, 250117 Shandong People’s Republic of China; 3 L.T.S.I., Inserm UMR 1099 - Université de Rennes, Campus de Beaulieu - Bat. 22, 35042 Rennes, France; 4 Jiangsu Provincial Joint International Research Laboratory of Medical Information Processing, Centre de Recherche en Information Biomédicale, Sino-Français (CRIBs), Rennes, France

**Keywords:** Respiratory-motion prediction, Adaptive linear regression, Gating signals, Patient-specific

## Abstract

**Background:**

This study aims to validate the effectiveness of linear regression for motion prediction of internal organs or tumors on 2D cine-MR and to present an online gating signal prediction scheme that can improve the accuracy of MR-guided radiotherapy for liver and lung cancer.

**Materials and methods:**

We collected 2D cine-MR sequences of 21 liver cancer patients and 10 lung cancer patients to develop a binary gating signal prediction algorithm that forecasts the crossing-time of tumor motion traces relative to the target threshold. Both 0.4 s and 0.6 s prediction windows were tested using three linear predictors and three recurrent neural networks (RNNs), given the system delay of 0.5 s. Furthermore, an adaptive linear regression model was evaluated using only the first 30 s as the burn-in period, during which the model parameters were adapted during the online prediction process. The accuracy of the predicted traces was measured using amplitude metrics (MAE, RMSE, and R^2^), and in addition, we proposed three temporal metrics, namely crossing error, gating error, and gating accuracy, which are more relevant to the nature of the gating signals.

**Results:**

In both 0.6 s and 0.4 s prediction cases, linear regression outperformed other methods, demonstrating significantly smaller amplitude errors compared to the RNNs (*P* < 0.05). The proposed algorithm with adaptive linear regression had the best performance with an average gating accuracy of 98.3% and 98.0%, a gating error of 44 ms and 45 ms, for liver cancer and lung cancer patients, respectively.

**Conclusion:**

A functional online gating control scheme was developed with an adaptive linear regression that is both more cost-efficient and accurate than sophisticated RNN based methods in all studied metrics.

**Supplementary Information:**

The online version contains supplementary material available at 10.1186/s13014-023-02341-1.

## Introduction

Gating technique involves synchronizing the delivery of radiation beams with specific phases of the patient's respiratory cycle to ensure precise targeting of the tumor while minimizing the dose to healthy tissues. The gating scheme relies on monitoring the patient's respiratory or motion signals, often through external markers or imaging modalities. These gating signals ensure that radiation is delivered only when the patient's motion is within the predetermined range, known as the “gating window” which is specific to each patient. Therefore, the accuracy of the gating signals plays a key role in the efficacy and reduction of side effects of the gating scheme to compensate for respiratory motions during radiotherapy [1, 2]. The recent advent of hybrid on-line magnetic resonance imaging (MRI)-guidance systems, represented by the Elekta Unity MR-Linac (Elekta AB, Stockholm, Sweden), provides higher accuracy in producing amplitude-based gating signals, as they directly visualization of tumors and internal structures without the need for implanted fiducials and/or external respiratory signals for target localization [3, 4].

Nevertheless, achieving real-time acquisition of 3D target volumes remains a challenge due to the trade-offs between spatial and temporal resolution. Previous studies reported that interleaved orthogonal slices (sagittal and coronal) can be obtained at adequate frequencies (4–8 Hz) for monitoring respiratory motion, enabling the reconstruction of the tumor's 3D position over time [5]. Amplitude-based gating performed superior to phase-based gating, as irregularities in breathing depth are better resolved when analyzing amplitudes instead of phases [6]. In MR-guided radiotherapy, the gating scheme for respiratory synchronization irradiation utilizes amplitude-based gating signals.

In order to compensate for the system delay of gating in Unity, it is vital to forecast the location of tumors and critical internal structures in real-time [7]. Klüter [8] reported latencies ranging from 300 to 436 ms for the Co60 version of the MRIdian using gating while Uijtewaal et al. [9] reported a latency of 330 ms for MLC-tracking on the Unity. Lamb et al. [10] however reported the overall system latency of gating was indicated to be within 0.5 s, including the image acquisition time, the contour-based target motion tracking and the beam on/off triggering. Thus, we fixed the latency of 0.5 s in our study to take into account the end-to-end system delay in the particular case of MRI guided gating application though it may not necessarily reflect the state-of-the-art for MRI-guided precision radiotherapy in the ideal scenario.

There are a number of alternative methods for predicting the trajectory of respiratory movements. Linear filters and their generalization have been largely used [11–13]. Probabilistic frameworks, including Bayesian inference [14], Kalman filters [15] and particle filters have also been widely proposed in several studies. More innovative and complex techniques such as support vector regression [11, 16], neural networks [12, 17, 18] and recurrent neural networks (RNN) [19, 20] have been explored for respiratory motion prediction and have demonstrated their effectiveness. In a recent review focusing on the primary prediction filters mentioned above, linear approaches were found to be sufficiently effective in prediction compared to more complex methods when using respiratory signals collected during Cyber-knife treatment [21]. However, it should be noted that directly translating prediction methods developed for respiratory signals guidance to MRI-guided treatments can be challenging due to the differences in imaging modalities (i.e., interleaved orthogonal slices instead of stereo X-ray images) and longer acquisition periods (e.g., 150–380 ms instead of 30 ms for X-ray fluoroscopy [22]), as well as overall system latencies (up to 500 ms [10]). Hence, it is crucial to validate the suitability of these prediction filters for internal organ or tumor position obtained from 2D cine-MR data, extending beyond just abdominal or thoracic amplitudes. Taking into account beam gating, an amplitude-based gating method was simulated in this study, where the beam is activated when the tumor is detected within “gating window”. Specifically, a binary [0,1] gating signal is generated to control the beam on/off by comparing the predicted tumor motion trajectory with the specified “gating window”.

**Fig. 1:**
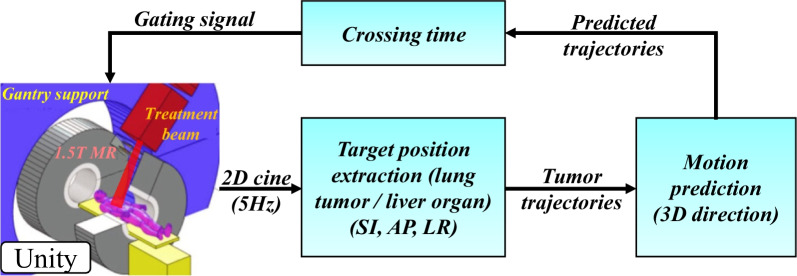
Flow chart of gating signals prediction for radiotherapy

In the current study, we focused on the comparison of linear models with three state-of-the-art deep RNN models, coupled with temporal metrics for the accuracy measure of the generated binary gating signal. Figure 1 shows the flow chart of the optimal gating signal prediction. Furthermore, we evaluated an adaptive regression model, in which the training on each patient’s online data lasted only for 30 s for the burn-in period and model parameters were updated during the online prediction process.

## Materials and methods

### Subjects

The study enlisted 21 patients with liver cancer and 10 with lung cancer, all receiving Elekta Unity radiation therapy from October 2020 to May 2023. Among these patients, 22 males and 9 females, averaging 62.1 years in age (ranging from 45 to 83), participated. 2D cine-MR images were acquired at the beginning of each radiotherapy session, with an acquisition duration ranging from 313 to 483 s.

### Data acquisition

Continuous MR images with 5 Hz imaging frequency were acquired by the Unity comprising an Achieva 1.5T MR scanner and a 7 MV flattening-filter-free linear accelerator. To strike a balance between acquisition time, signal-to-noise ratio, and resolution, we opted for an acquisition frequency of 5 Hz, which is considered to be reasonable [23]. The spatial resolution of 2D cine images is 3 × 3 mm^2^. The MRI-Linac allows real-time acquisition of three orthogonal planes (coronal, sagittal, and transverse), with motion in the left–right (LR) and superior-inferior (SI) directions measured from coronal slices, and motion in the anterior–posterior (AP) direction derived from sagittal slices.

**Fig. 2:**
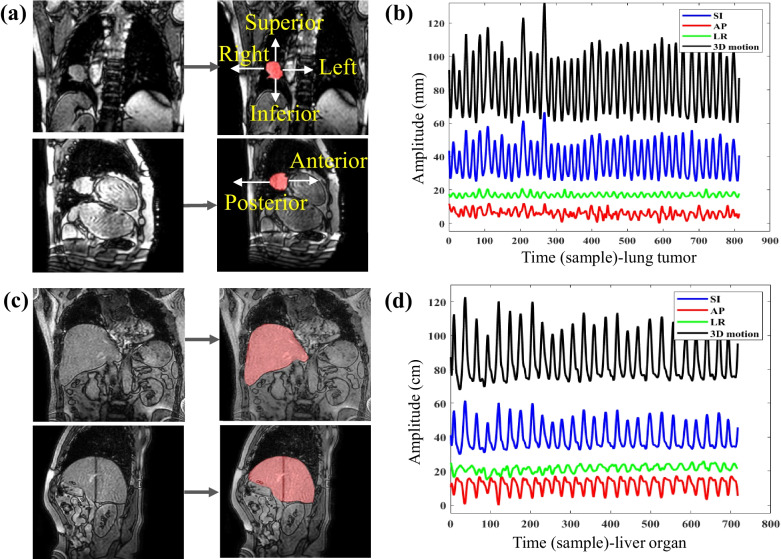
**a** Labeled lung tumor in coronal and sagittal slices. **b** Tumor centroid trajectories in three directions and 3D total motion. **c** Labeled liver organ in coronal and sagittal slices. **d** Liver centroid trajectories in three directions and 3D total motion

Unlike the MRI sequences for lung cancer patients, the movement of the liver organ's is considered instead of that of the tumor since liver is not a hightly compressible organ while the tumor borders are extremely difficult to identify in practice. The gross tumor volumes (GTVs) of lung patients (Fig. 2a) and liver organ of liver patients (Fig. 2c) were outlined by an experienced radiologist, and then their trajectories (Fig. 2b, d) in three directions of the tumor/liver boundary were extracted from the 2D cine-MR. In amplitude-based gated radiotherapy, we combine the motion from these three different directions into a Euclidean distance called “3D total motion”. This approach computes SI, AP, and LR displacements as the length of a three-dimensional vector, ensuring a holistic consideration of motion. And it allows specific gating thresholds to be set based on the amplitude of 3D total tumor motion for each patient, helping to simplify treatment planning and reduce motion-related complexity. Table 1 shows the mean amplitude of motion and the min–max range (in millimeters), along with the mean respiratory cycle and the min–max range (in seconds) for liver organ and lung tumors.

**Table 1:** Movement characteristics of liver organs and lung tumors

	SI (mm)	AP (mm)	LR (mm)	3D motion (mm)	Period (s)
Liver organ	21.3 (5.6–40.1)	7.9 (3.2–13.1)	9.3 (4.3–15.9)	25.5 (14.5–43.2)	4.6 (2.9–7.4)
Lung tumor	16.2 (3.1–25.2)	4.2 (2.8–5.9)	2.8 (0.9–4.5)	17.2 (5.1–25.5)	3.4 (2.4–5.4)

Mean amplitude of motion and (min–max) range in millimeters, and mean respiratory cycle and (min–max) range in seconds

*SI* superior Inferior, *LR* left right, *AP* anterior posterior

### Data interception

Trajectory of each patient was divided into training set (70%) and testing set (30%), and 20% of training set was a validation set to cross validate and monitor the performance of training and the optimization of the hyper-parameters for Ridge regression, L2–L1 regression and RNN models. The inputs and outputs of the predictor were segmented with a sliding window consisting of one pair of input and output data, which were denoted as ${x}_{i}$ and ${y}_{i}$. The successive input ${x}_{i+1}$ was generated by moving the sliding window by 1 sample forward, and the sliding window was moved forward until the last available observation in the training part was hit. The length of each ${x}_{i}$ represents the number of samples used to make prediction, we fixed $m=15$ (about 3 s) in this study. The length of ${y}_{i}$ is determined by the prediction window. Since the sampling period is 0.2 s, ${y}_{(i,1)}$, ${y}_{(i,2)}$, ${y}_{(i,3)}$ represent the prediction windows of 0.2, 0.4 and 0.6 s, respectively. In this study, since the system delay is 0.5 s while the MRI sampling period is 0.2 s, we are confronted with the choice of either 0.4 s (under prediction) or 0.6 s (over prediction) for the prediction window.

### Gating signals prediction algorithm

#### Linear predictors

The linear regression models assume the linear relationship between the future data (${\widehat{y}}_{(i,j-1)}$ and ${\widehat{y}}_{(i,j)}$) and the past available data ${x}_{i}$:

1 $$\left\{\begin{array}{c}{\widehat{y}}_{\left(i,j-1\right)}={x}_{i}^{T}{\beta }_{j-1}\\ {\widehat{y}}_{\left(i,j\right)}={x}_{i}^{T}{\beta }_{j}\end{array}\right.$$

where $T$ denotes the transpose, the coefficient vector defined as ${\beta }_{j}=({\beta }_{j1}, \dots , {\beta }_{jm})$ for $m=15$ and $j=2$ for prediction window of 0.4 s or $j=3$ for prediction window of 0.6 s, note that this rule applies to all $j$ in the following.

The loss function of linear regression is to minimize the sum of the squares of the residuals, given by

2 $$\left\{\begin{array}{c}L({\beta }_{j-1})=\sum_{i=1}^{N}{({y}_{\left(i,j-1\right)}-{x}_{i}^{T}{\beta }_{j-1})}^{2}\\ L({\beta }_{j})=\sum_{i=1}^{N}{({y}_{\left(i,j\right)}-{x}_{i}^{T}{\beta }_{j})}^{2}\end{array}\right.$$

For ridge regression, the loss function to be minimized is the penalized residual sum of squares [24]:

3 $$\left\{\begin{array}{c}L({\beta }_{j-1})=\sum_{i=1}^{N}{({y}_{\left(i,j-1\right)}-{x}_{i}^{T}{\beta }_{j-1})}^{2}+{\lambda \Vert {\beta }_{j-1}\Vert }_{2}^{2}\\ L\left({\beta }_{j}\right)=\sum_{i=1}^{N}{\left({y}_{\left(i,j\right)}-{x}_{i}^{T}{\beta }_{j}\right)}^{2}+{\lambda \Vert {\beta }_{j}\Vert }_{2}^{2}\end{array}\right.$$

where the optimal parameter $\lambda$ is implemented by Leave-One-Out cross-validation (LOOCV). The regularization term shrinks the magnitude of the coefficient vector $\beta$, which leads to a reduction of the noise level of the prediction signals [25].

For the L2–L1 regression, the l1-norm of the parameters is used as the penalty term in the loss function [26]:

4 $$\left\{\begin{array}{c}L({\beta }_{j-1})=\sum_{i=1}^{N}{({y}_{\left(i,j-1\right)}-{x}_{i}^{T}{\beta }_{j-1})}^{2}+\lambda {\Vert {\beta }_{j-1}\Vert }_{1}\\ L\left({\beta }_{j}\right)=\sum_{i=1}^{N}{\left({y}_{\left(i,j\right)}-{x}_{i}^{T}{\beta }_{j}\right)}^{2}+\lambda {\Vert {\beta }_{j}\Vert }_{1}\end{array}\right.$$

where the optimal $\lambda$ is chosen by the generalized cross-validation (GCV) [27], and the optimal solution $\beta$ is obtained by the alternating direction method of multipliers (ADMM) [28].

Due to the lower complexity of linear regression methods, we also implemented and evaluated the adaptive linear regression with a burn-in period of the first 30 s (150 samples) for each patient. The minimization of $\mathrm{L}(\upbeta )$ is performed continuously to update the model parameters (${\beta }_{j-1}$ and ${\beta }_{j}$) with the incoming data of the same patient.

#### RNN models

RNNs capture temporal dependencies in data by processing sequences, making them useful for predicting respiratory motion. Gradient vanishing and explosion caused RNNs to lose their grasp on nonlinear relationships. Solutions include long short-term memory (LSTM), bidirectional LSTM (Bi-LSTM), and gated recurrent unit networks (GRUs). Detailed description of the LSTM, Bi-LSTM and GRU and their performance for predicting respiratory motion can be found elsewhere [19, 20, 29].

A validation set was used to cross validation to monitor the performance of training and search the optimal hyper-parameters. During the training phase, the Adaptive Moment Estimation (Adam) optimizer was used to optimize the loss function due to its adaptability to varying learning rates, effective handling of sparse gradients common, robustness to noisy data, and efficient parameter updates [19]. The following hyperparameters were chosen based on a search conducted by Lombardo et al. [30]: the number of layers from {1, 3, 5, 10}, dropout rates from {0, 0.1, 0.2}, learning rates from {0.0001, 0.0005, 0.001, 0.005, 0.01}, and batch sizes from {16, 32, 64, 128}.

#### Crossing time

“Crossing time” refers to the moment when the tumor's position crosses a predefined threshold. When the tumor crosses below the predefined threshold, referred to as ‘crossing-on’, the radiation beam is activated. Conversely, when the tumor crosses above the thresh-old, termed as ‘crossing-off’, the radiation beam is deactivated. Since the sampling frequency of MR images is 5 Hz, it is reasonable to assume that the shape of the respiratory curve can be restored by connecting each frame of the continuous images. Therefore, we proposed a linear interpolation to predict the threshold crossing time:

5 $${\widehat{T}}_{cross}= \frac{Th-{\widehat{y}}_{(i,j)}}{{\widehat{y}}_{(i,j)}-{\widehat{y}}_{(i,j-1)}}\times\Delta T+{T}_{i}+j\times\Delta T$$

where ${T}_{i}$ is the current sample time, ${\widehat{y}}_{(i,j-1)}$ and ${\widehat{y}}_{(i,j)}$ the predictions by Eq. 1, $\Delta T= 0.2\mathrm{ s}$ the MRI sampling period, and $Th$ the threshold, set as the average of the respiratory trajectory in the SI direction during the burn-in period for simplicity. Note that a lower threshold means beaming during the more stagnant phase of exhalation and thus ensures better treatment margins while increasing the overall treatment time. The optimal trade-off between radiation precision and efficiency is beyond the scope of the present study. The reference crossing time (gold standard) could be calculated in a similar manner with Eq. 5, using ${y}_{(i,j-1)}$ and ${y}_{(i,j)}$ instead of their predictions.

#### Gating signals generation

**Fig. 3:**
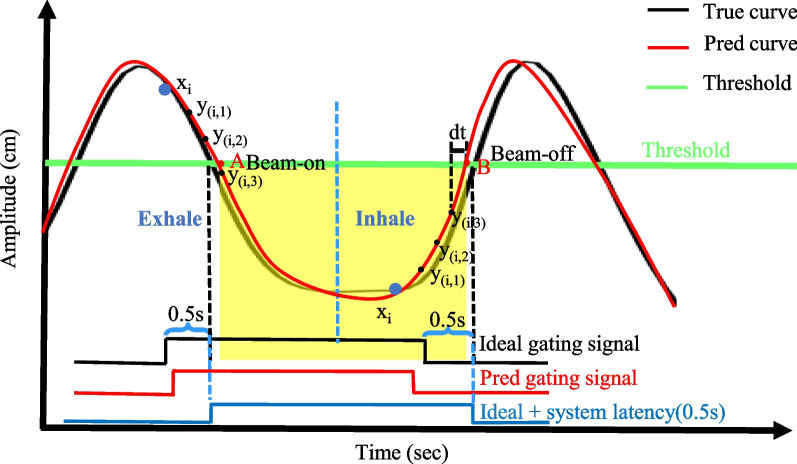
Gating control scheme by thresholding the predicted tumor trajectory for prediction window of 0.6 s

Figure 3 shows the gating control scheme by thresholding the predicted tumor trajectory for prediction window of 0.6 s. The system latency was 0.5 s in this study, thus ideal gating signals should be 0.5 s earlier than ideal crossing time. In the experiment, taking the 0.6 s prediction window $(j = 3)$ as an example, there are two conditions for triggering gating: (1) when the motion curve crosses the threshold between ${y}_{(i,2)}$ and ${y}_{(i,3)}$ (point A in Fig. 3), or (2) when the time difference ($dt$) between ${\widehat{y}}_{(i,3)}$ and cross time is less than 0.1 s (point B in Fig. 3). For the prediction window of 0.4 s ($j=2$), the decision condition is changed from ${y}_{(i,2)}$ and ${y}_{(i,3)}$ to ${y}_{(i,1)}$ and ${y}_{(i,2)}$. The black and red binary control signals were generated by the reference and predicted crossing time, respectively. Additional file 1: Table S1 describes in detail the process of adaptive gating signals generation with an ever-increasing training data set.

### Performance evaluation

#### Amplitude metrics

The mean absolute error (MAE) is the mean absolute difference between the predicted and observed values defined by:

6 $$MAE=\frac{1}{N}\sum_{i=1}^{N}\left|{y}_{i}-{\widehat{y}}_{i}\right|$$

where ${y}_{i}$ and ${\widehat{y}}_{i}$ are the actual and predicted respiration data respectively, and $N$ the number of total points.

The root mean square error (RMSE) is a measure of accuracy, to compare forecasting errors of different models for a particular dataset, and is defined as:

7 $$RMSE= \sqrt{\frac{1}{N}\sum_{i=1}^{N}{\left({y}_{i}-{\widehat{y}}_{i}\right)}^{2}}$$

The coefficient of determination ${R}^{2}$, is the proportion of the variation in the dependent variable that is predictable from the independent variable(s):

8 $${R}^{2}=1-\frac{\sum_{i=1}^{N}{\left({y}_{i}-{\widehat{y}}_{i}\right)}^{2}}{\sum_{i=1}^{N}{\left({y}_{i}-{\overline{y} }_{i}\right)}^{2}}$$

#### Temporal metrics

For gated radiotherapy, it is essential in predicting accurately the beam on/off control when the target crosses threshold position rather than predicting the respiratory curve itself. We thus proposed the gating on/off error and gating accuracy to compare with the ideal gating signals.

We first define the crossing on/off error as the temporal difference of threshold-crossing between the true and predicted curves in both on/off directions. The gating on/off error, on the other hand, denotes the temporal difference of beam-on/off control between the ideal (0.5 s in advance of the real threshold-crossing) and the predicted gating signals.

The gating accuracy is the ratio of overlapped duration (of the ideal and predicted gating signals) over the entire therapy:

9 $${\text{Gating}}\;{\text{accuracy}} = \left[ {1 - \frac{{\mathop \sum \nolimits_{{{\text{i}} = 1}}^{{\text{N}}} \left( {{\text{Tgaterr}}} \right)}}{{{\text{Total}}\;{\text{time}}\;{\text{of}}\;{\text{therapy}}}}} \right] \times 100\%$$

where $N$ is the number of breathing cycles during therapy of each patient and $\mathrm{Tgaterr}$ the non-overlapping period of the ideal and predicted gating signals.

#### Statistical tests

We performed a Wilcoxon signed-rank test to analyze whether there is a statistically significant difference between the metrics (R^2^ and Gating accuracy) obtained from the different models on the testing set. A *p* value of less than 0.05 was considered significant.

## Results

### Linear versus RNN regression

Linear regression with and without its regularizations (Ridge and L2-L1) variants are compared to three classical RNN models and Kalman filter [15] for prediction performance evaluations. Since RNN requires a large amount of training data, we first use 70% of the data (more than 4 min) in these six models for training and the rest for testing.

Table 2 shows the mean and standard deviation of amplitude errors (MAE, RMSE, and R^2^) for different methods with 0.4 s and 0.6 s prediction window for liver organs of 21 liver cancer patients and lung tumors of 10 lung cancer patients. No matter the choice of prediction length, linear regression without regularization performed the best, followed by the Ridge, and linear methods have significantly smaller amplitude errors than those of the RNNs (*P* < 0.05), not to mention the unrealistic 70–30% data partition adopted in favor of the RNNs. Meanwhile, the magnitude-based results demonstrated the excellent predictive power of the linear regression for motion prediction of both liver organ and lung tumors.

**Table 2:** Mean and std deviation of amplitude errors with 0.4 s and 0.6 s prediction window for 21 liver cancer patients and 10 lung cancer patients

PW	Model	Liver organs			Lung tumors		
		MAE (mm)	RMSE (mm)	R^2^ (None)	MAE (mm)	RMSE (mm)	R^2^ (None)
0.4 s (j = 2)	**Linear**	**0.60 ± 0.15**	**1.61 ± 0.64**	**0.96 ± 0.03**	**0.86 ± 0.56**	**1.84 ± 0.84**	**0.97 ± 0.02**
	Ridge	0.60 ± 0.15	1.61 ± 0.64	0.96 ± 0.03	0.90 ± 0.60	1.87 ± 0.87	0.97 ± 0.02
	L2-L1	1.26 ± 0.33	2.16 ± 0.54	0.94 ± 0.04	1.41 ± 0.38	2.35 ± 0.66	0.96 ± 0.01
	LSTM	1.87 ± 0.38	2.94 ± 0.56	0.87 ± 0.12	2.51 ± 1.39	3.93 ± 1.67	0.92 ± 0.03
	Bi-LSTM	1.70 ± 0.63	2.85 ± 0.75	0.89 ± 0.11	2.26 ± 0.99	3.57 ± 1.09	0.93 ± 0.02
	GRU	1.58 ± 0.46	2.69 ± 0.60	0.90 ± 0.08	2.18 ± 0.92	3.46 ± 1.04	0.94 ± 0.02
	KF	2.07 ± 0.48	2.66 ± 0.62	0.95 ± 0.03	2.34 ± 0.83	2.77 ± 0.95	0.93 ± 0.03
0.6 s (j = 3)	**Linear**	**1.19 ± 0.40**	**1.55 ± 0.51**	**0.97 ± 0.02**	**1.65 ± 0.87**	**2.11 ± 1.06**	**0.97 ± 0.03**
	Ridge	1.2 ± 0.39	1.56 ± 0.50	0.97 ± 0.02	1.69 ± 0.92	2.16 ± 1.12	0.97 ± 0.03
	L2-L1	2.23 ± 0.71	2.85 ± 0.90	0.91 ± 0.06	2.52 ± 0.71	3.29 ± 0.96	0.94 ± 0.03
	LSTM	1.87 ± 0.38	2.94 ± 0.56	0.87 ± 0.12	3.32 ± 1.40	4.36 ± 1.97	0.91 ± 0.04
	Bi-LSTM	1.70 ± 0.63	2.85 ± 0.75	0.89 ± 0.11	3.39 ± 1.64	4.56 ± 2.27	0.89 ± 0.07
	GRU	1.58 ± 0.46	2.69 ± 0.60	0.90 ± 0.08	3.50 ± 1.48	4.62 ± 1.98	0.88 ± 0.06
	KF	2.55 ± 0.99	3.41 ± 1.21	0.89 ± 0.01	2.89 ± 1.13	3.60 ± 1.32	0.87 ± 0.03

The best performing model is shown in bold for each prediction window

*PW* prediction window, *KF* Kalman filter

Table 3 shows the mean and standard deviation of gating errors with prediction length of 0.4 s and 0.6 s for liver organs of 21 liver cancer patients and lung tumors of 10 lung cancer patients, as well as calculation time for each model. For the gating accuracy, choosing over-prediction (0.6 s) is significantly better than under-prediction (0.4 s), all factors being equal otherwise (*P* < 0.05). When the prediction window is 0.6 s, the linear regression achieved the best performance with an average gating accuracy of 98.3% and 98.0%, a gating error of 56 ms and 45 ms, for liver cancer and lung cancer patients, respectively. Table 4 shows the *P* values obtained from Wilcoxon signed-rank test pairwise model comparisons with prediction length of 0.6 s. Performances of the linear regression are significantly higher than those of RNNs in both amplitude metrics (R^2^) and temporal metrics (Gating accuracy) (*P* < 0.05). To address the potential for overfitting in linear regression and ridge regression, we performed cross-validation on each respiratory curve. Although the temporal correlations within the time series data could potentially affect the cross-validation results, it is noteworthy that linear model achieved an average gating accuracy of 98.2% and 97.7% for liver cancer and lung cancer patients, respectively.

**Table 3:** Mean and std deviation of gating errors with prediction length of 0.4 s and 0.6 s for 21 liver cancer patients and 10 lung cancer patients, as well as calculation time for each model

PW	Model	Liver organs			Lung tumors			Time (ms)
		Crossing (ms)	Gating (ms)	Gat-acc (%)	Crossing (ms)	Gating (ms)	Gat-acc (%)	
0.4 s (j = 2)	Linear	30 ± 25	190 ± 27	93.9 ± 2.1	25 ± 28	183 ± 65	91.6 ± 3.3	0.06
	Ridge	32 ± 27	190 ± 30	93.8 ± 2.1	25 ± 29	184 ± 66	91.4 ± 3.3	0.07
	L2-L1	58 ± 24	184 ± 46	93.3 ± 2.3	44 ± 29	191 ± 76	90.1 ± 3.9	0.03
	LSTM	75 ± 64	232 ± 141	93.0 ± 4.2	75 ± 52	233 ± 111	90.5 ± 4.7	3.71
	Bi-LSTM	73 ± 53	285 ± 261	90.6 ± 9.9	70 ± 52	267 ± 101	90.0 ± 4.8	5.43
	GRU	71 ± 69	283 ± 250	90.5 ± 16	47 ± 44	249 ± 107	90.2 ± 4.7	3.36
	KF	68 ± 32	240 ± 130	91.2 ± 17	52 ± 32	212 ± 94	91.2 ± 3.7	0.02
0.6 s (j = 3)	Linear	56 ± 33	56 ± 33	98.3 ± 1.0	45 ± 30	45 ± 30	98.0 ± 1.8	0.07
	Ridge	58 ± 33	58 ± 33	98.2 ± 1.1	47 ± 31	47 ± 31	97.8 ± 2.5	0.06
	L2-L1	80 ± 50	80 ± 50	97.2 ± 1.2	80 ± 47	80 ± 47	96.4 ± 2.5	0.02
	LSTM	112 ± 85	112 ± 85	96.1 ± 2.6	138 ± 74	138 ± 74	94.7 ± 3.3	4.36
	Bi-LSTM	130 ± 101	130 ± 101	96.0 ± 2.7	131 ± 73	131 ± 73	94.7 ± 4.0	7.25
	GRU	112 ± 72	112 ± 72	96.5 ± 2.3	125 ± 80	125 ± 80	95.1 ± 3.4	4.32
	KF	102 ± 36	102 ± 36	96.9 ± 1.2	115 ± 70	115 ± 70	95.2 ± 1.9	0.02
	**AL (30 s)**	**44 ± 23**	**44 ± 23**	**98.3 ± 0.6**	**45 ± 31**	**45 ± 31**	**98.0 ± 1.7**	**0.99**

The best performing model for gating errors is shown in bold.

*PW* prediction window, *Crossing* crossing on/off error, *Gating* gating on/off error, *Gat-acc* gating accuracy, *KF* Kalman filter, *AL* adaptive linear, *30 s* using only the first 30 s as the burn-in period.

**Table 4:** *P* values obtained from Wilcoxon signed-rank test pairwise model comparisons with prediction length of 0.6 s

Model 1	Model 2	R^2^	Gat-acc
Linear	Ridge	0.0001	0.0005
	L2-L1	0.0001	0.0001
	LSTM	0.0001	0.0001
	Bi-LSTM	0.0001	0.0001
	GRU	0.0001	0.0001
	KF	0.0001	0.0001
Adaptive Linear (30 s)	Linear	0.5663	0.5200
	Ridge	0.0013	0.0580
	L2-L1	0.0010	0.0009
	LSTM	0.0010	0.0001
	Bi-LSTM	0.0010	0.0001
	GRU	0.0010	0.0001
	KF	0.0001	0.0001

A *P* value of less than 0.05 was considered significant

*Gat-acc* gating accuracy, *30 s* using only the first 30 s as the burn-in period, *KF* Kalman filter

### Adaptive regression

Due to the lower complexity of linear regression methods, we also implemented and validated the approach of training the model with the first 30 s (150 samples) for each patient as the burn-in period and keep adapting the model parameters with the incoming data during the prediction and gating signals generation process. As shown in Table 3, the proposed framework with adaptive linear regression achieved convincing performances in real clinical scenarios with an average gating accuracy of 98.3% and 98.0%, a gating error of 44 ms and 45 ms, for liver cancer and lung cancer patients, respectively. The predictive performance of adaptive linear regression is not significantly different from that of non-adaptive linear regression using more than 4 min of training data (*P* > 0.05, see in Table 4).

### Calculation time

All experiments were conducted using the Keras API with the TensorFlow backend and were executed on an Intel 4-core 2.4-GHz CPU, a NVIDIA GeForce GTX1660 Ti GPU, 512 GB SSD and 20 GB RAM machine. The time required to predict the gating signals was calculated for all methods mentioned above (see Table 3). For the non-adaptive mode, the calculation time of linear models are less than 0.1 ms and the calculation time of RNN models are between 3 and 8 ms. For the adaptive mode, the most time-consuming part was the update of the linear regression predictor, requiring an average of 1 ms, the rest being identical to the non-adaptive mode, i.e. less than 0.1 ms.

## Discussion

The study introduced a linear interpolation for the prediction of the threshold-crossing time and achieved better temporal accuracy in the subsequent gating signals. A fixed online linear model was first used to predict tumor locations for 0.4 s and 0.6 s ahead and to update the predicted threshold-crossing time, while the gating signal was triggered 0.5 s (system delay) ahead of the predicted crossing time. Moreover, this study gave the certain criteria in temporal metrics. For the best performance, the error is 0, it is proved that the predicted gating signal is equal to the ideal gating signal. For dummy performance, the maximum error was latency time (0.5 s) even without predicting, and here gating time is equal to crossing time. Indeed, we observe identical crossing and gating errors in the case of over-prediction whereas in the case of under-prediction crossing errors seem to be reduced thanks to its higher precision, gating errors are getting worse since the gating decisions are made typically between 0.2 s and 0.4 s in advance with a system delay of 0.5 s. We thus conclude that over-prediction is a preferable choice compared to under-prediction when system delay is not a multiple of the MRI sampling period.

**Fig. 4:**
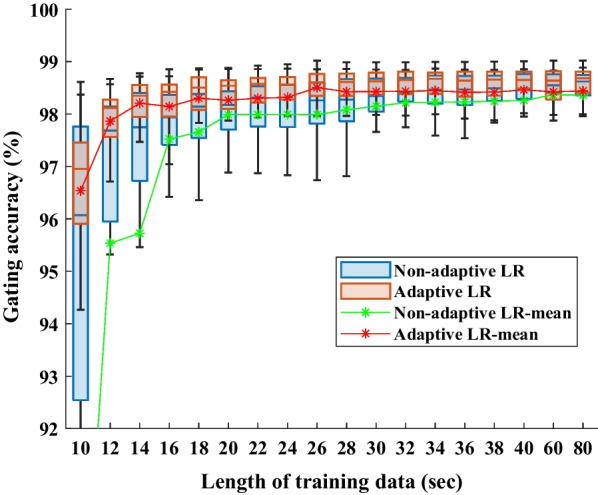
Gating accuracy using adaptive/non-adaptive linear regression with different burn-in period

To verify the minimum number of training data required to train a stable linear regression model, we tested its adaptive and non-adaptive versions with different lengths of training data. Figure 4 shows the gating accuracy using adaptive and non-adaptive linear regression with different sizes of training data. At the beginning, the gating accuracy increased with the increase of training data, the average gating accuracy of the adaptive version reached 98% at 14 s and tended to increase slowly, while the non-adaptive version reached the stable prediction ability at 32 s. When the training data reached 80 s, the prediction ability of the adaptive version and the non-adaptive version are the same. According to the box plot, when the training data is less than 40 s, the gating accuracy of the adaptive version is obviously better than that of the non-adaptive version. For the patient-specific prediction model, the adaptive version can significantly reduce the burn-in time.

As observed in several studies [5, 9, 15, 20], the predictive performance decreased with increasing forecasted time span. However, for the 0.4 s and 0.6 s prediction windows, linear regression still achieved sub-resolution accuracy (RMSE < 2.5 mm). The good performance of the linear regression for the 0.6 s prediction window shows that it can successfully account for the system latency found by Glitzner et al. [4] when performing MLC tracking on the Elekta Unity MR-linac. As shown in Table 1, the range of respiratory cycles for the 10 lung cancer patients was 2.4–5.4 s and the range of 3D movement amplitudes was 5.1–25.5 mm; the range of respiratory cycles for the 21 liver cancer patients was 2.9–7.4 s and the range of 3D movement amplitudes was 14.5–43 mm. These large ranges show that there are large differences in respiratory movements between each patient and that respiratory movements are patient-specific. Therefore, we proposed to use the first 30 s (burn-in period) of treatment for each patient to train a patient-specific adaptive linear model, which is used for real-time prediction of tumor location during subsequent radiotherapy for that patient and for gating signal generation. Based on the excellent experimental results, the patient-specific online gating signal prediction scheme based on the linear regression model proposed in this study can be widely applied in MRI-guided radiotherapy for lung and liver cancer.

A recent review study by Jöhl et al. [21] found that a continuously re-optimized (i.e., online) linear regression model performed best on average compared to other motion predictors such as artificial neural networks or Kalman filters. Sharp et al. [15] note the relatively worse performance of the Kalman filter predictor when compared with linear and ANN predictors. In this study, we also compared the traditional methods represented by Kalman filtering with linear regression. Interestingly, our findings consistently indicate that linear regression outperforms Kalman filtering. This may reflect the difficulty in estimating the state transition matrix from such a small amount of data.

Our experimental results on small sample sets verified the excellent performance of linear regression, considerably superior to that of the RNN. The major drawback of classic RNNs is that they are notoriously difficult to train. One important consideration that we addressed within this study is the effect of the non-stationary nature of breathing. Anetai et al. [31] clearly illustrated that the movement and pattern of breathing can easily change individually various. They developed and verified novel respiratory criteria for selecting optimal breathing for gating radiation treatment and defining numerical targets for respiratory gating. For the adaptive linear regression in the study, the predictive parameters were adjusted in real time based on the most recent tumor motion, which may address the issue of respiratory pattern variability. In the current experiment, despite the adaptive regression model continuously increasing its training samples, the average calculation speed is still less than 1 ms, as patients’ radiotherapy time on Unity is between 15–20 min.

Quantifying liver tumor motion is difficult because of the difficulty of imaging free-breathing tumors, the difficulty of delineating tumors, and the complexity of tumor motion: rigid (translation and rotation), non-rigid, and the combination of both types of motion [32]. The liver's most significant movement typically occurs along the SI direction, influencing motion in the AP and LR directions. While liver tissue isn't highly compressible, some degree of deformation is expected [33]. As liver tumors often reside within a larger liver volume, the extent of tumor displacement during the respiratory cycle depends on its specific location within the liver [34]. Prior research has indicated a strong correlation between tumor motion within the liver and the concurrent movements of the liver organ, its vasculature, and the diaphragm [35–38]. Yang et al. [36] highlighted that the consistency of tumor and diaphragm motion varies based on the distance separating them. Zhao et al. [34] employed a five-lobe classification scheme to subdivide the liver, noting that segments within distinct liver lobes display differing levels of motion. Consequently, when pinpointing the region of interest (ROI) for a tumor, it's important to not only encompass the tumor but also consider adjacent boundaries, neighboring organs, or any area anticipated to move in synchrony with the target [23]. The precise selection of the ROI representing tumor motion is of paramount importance and should be determined by experienced professionals based on the specific clinical context. In this study, to overcome the difficulties in liver tumor contouring on 2D cine-MR without the use of contrast agents and based on the high correlation properties of the two motions, we admittedly simplified the tracking problem to deal with the organ centroids in order to validate the proposed prediction system. It is beyond the scope of this study as to how to accurately select the ROI representing the tumor motion and it's tracking methods.

What’s more, the manual labelling of liver organs and tumors from 2D cine-MR is time-consuming and only valid for the current proof-of-concept study while not compatible with a real-time clinical application scenario. For the future work, we aim to combine automatic tumor localization and prediction to evaluate the potential dosimetric improvements. MRiPT (MR-guided proton therapy) has progressed significantly with the development of clinical prototypes expected in 5–10 years. It will need a dedicated workflow similarly to Unity, and gated treatments could be expected as the default treatment style [39–41]. Therefore, our proposed gating signals prediction algorithm is expected to be used for proton therapy in the future and will have better clinical application value.

In conclusion, the proposed algorithm could offset the system delays in beaming on/off switching and thus deliver the dose with better temporal accuracy. The Unity is potentially capable of performing more accurate radiotherapy procedures when coupled with the proposed gating signals prediction algorithm.

### Supplementary Information

**Additional file 1. **Algorithmic steps for generating adaptive gating signals.

## Data Availability

The Unity database generated and analyzed during the current study are available from the corresponding author B. Li (Email: bsli@sdfmu.edu.cn). The data are not publicly available for patient privacy purposes.
