# Supplementary material for: Online prediction for respiratory movement compensation: a patient-specific gating control for MRI-guided radiotherapy
Source: Radiat Oncol. 2023 Sep 11;18:149. doi: 10.1186/s13014-023-02341-1 (PMC10496354; doi:10.1186/s13014-023-02341-1)
Supplement: Supplementary file 1 — Additional file 1. Algorithmic steps for generating adaptive gating signals. [file 13014_2023_2341_MOESM1_ESM.docx]

| **Algorithm 1** Adaptive gating signal prediction |  |  |
| --- | --- | --- |
| **Training part (for burn-in):**  Get X, Y ∈ training data  Initialize $\beta_{j-1}, \beta_{j}$  **Testing part:**  **Input:** tumor traces $[x_{1}, . . ., x_{N} ]$  **Output:** gating signal $[T_{on}, T_{off}]$  1: $\mathrm{lookforbeamon}\leftarrow1$  2: $\mathbf{for}i\leftarrow1 to N\mathbf{do}$  3: $\left[ \hat{y}_{j-1}, \hat{y}_{j} \right]\boldsymbol{\leftarrow}\mathrm{pred} \boldsymbol{(}x_{i}\boldsymbol{,}\beta_{j-1}, \beta_{j}\boldsymbol{)}$ ⊳ see in Eq.1  4: $\mathrm{dt}\leftarrow T_{cross}-(T_{i}+j\times\Delta T)$ ⊳$T_{cross}$ see in Eq.5  5: $\mathbf{if}lookforbeamon = 1\mathbf{then}$  6: $\mathbf{if}condition 1\mathbf{== true then}$  7: $\mathrm{push} (T_{cross}- \mathrm{latency}) \mathrm{into} T_{on}$ ⊳ latency = 0.5sec  8: $\mathrm{lookforbeamon} \boldsymbol{\leftarrow} 0$  9: $\mathbf{end if}$  10: $\mathbf{else if}lookforbeamon=0\mathbf{then}$  11: $\mathbf{if}condition 2\mathbf{== true then}$  12: $\mathrm{push} (T_{cross}- \mathrm{latency}) \mathrm{into} T_{off}$  13: $\mathrm{lookforbeamon}\boldsymbol{\leftarrow} 1$  14: $\mathbf{end if}$  15: $\mathbf{end if}$  16: Update training data  17: Update $\beta_{j-1}, \beta_{j}$  18: $\mathbf{end for}$  19: $\mathbf{return} T_{on},T_{off}$ |  |  |
| ⊳ Condition 1: ${\boldsymbol{(}\hat{\boldsymbol{y}}}_{\boldsymbol{j}}\boldsymbol{>Th}\boldsymbol{and dt \leq0.5\Delta T) or (}{\boldsymbol{(}\hat{\boldsymbol{y}}}_{\boldsymbol{j-1}}\boldsymbol{>Th\geq}{\hat{\boldsymbol{y}}}_{\boldsymbol{j}}\mathbf{)}$⊳ Condition 2: ${\boldsymbol{(}\hat{\boldsymbol{y}}}_{\boldsymbol{j}}\boldsymbol{<Th}\boldsymbol{and dt \leq0.5\Delta T) or (}{\boldsymbol{(}\hat{\boldsymbol{y}}}_{\boldsymbol{j-1}}\boldsymbol{<Th\leq}{\hat{\boldsymbol{y}}}_{\boldsymbol{j}}\mathbf{)}$ | | |

Table S1. Algorithmic steps for generating adaptive gating signals
